# Supplementary material for: Causal Relationship between Aging and Anorexia Nervosa: A White-Matter-Microstructure-Mediated Mendelian Randomization Analysis
Source: Biomedicines. 2024 Aug 16;12(8):1874. doi: 10.3390/biomedicines12081874 (PMC11351342; doi:10.3390/biomedicines12081874)
Supplement: Supplementary file 1 [file biomedicines-12-01874-s001.zip › Supplementary Figure/Figure S8/Leaveoneout plot of MR analysis for TL to ubm-b-1871.pdf]

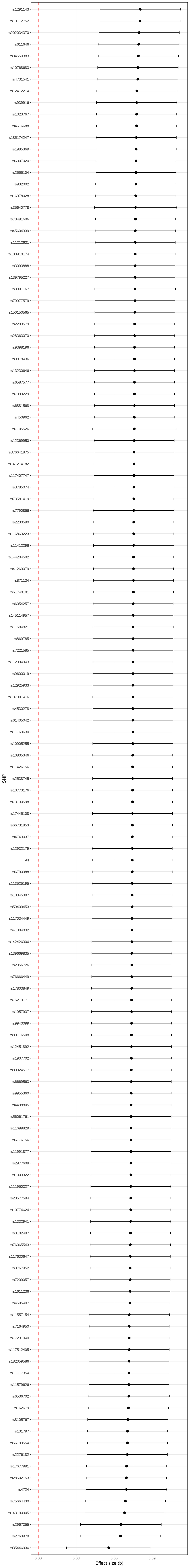

rs1291143
rs10112752
rs202034370
rs611646
rs34550383
rs10768683
rs4731541
rs12412214
rs939916
rs1023767
rs4616688
rs185174247
rs1985369
rs6007020
rs2555104
rs932002
rs16978028
rs35640778
rs78491606
rs45604339
rs11212631
rs188918174
rs3093888
rs139795227
rs3891167
rs79977579
rs150150565
rs2293579
rs28363070
rs9398196
rs9878436
rs13230646
rs6587577
rs7099229
rs6881568
rs450962
rs7705526
rs12369950
rs376641875
rs141214782
rs117407747
rs3785074
rs73581419
rs7790856
rs2230590
rs116863223
rs11412296
rs144204502
rs41269079
rs871134
rs61748181
rs6054257
rs145114957
rs11584821
rs869785
rs7221585
rs112394943
rs9600019
rs12925933
rs137901416
rs4530278
rs61405042
rs11769630
rs10905255
rs10805346
rs11426156
rs2538745
rs10773176
rs73730598
rs17445108
rs66731853
rs4743037
rs12932179
All
rs6790988
rs113525195
rs10845387
rs59409453
rs117034449
rs41304832
rs142426306
rs139669835
rs2056726
rs76666449
rs17803849
rs76219171
rs1957937
rs9940099
rs80116508
rs12451892
rs1907702
rs80324517
rs6669563
rs9955360
rs4498805
rs56061761
rs11699829
rs6776756
rs11991877
rs2977608
rs1003322
rs111950327
rs28577594
rs10774624
rs1332941
rs8102497
rs76065543
rs117630647
rs3767952
rs7209057
rs1611236
rs4695407
rs11557154
rs7164950
rs77231040
rs117512405
rs182059586
rs11117354
rs11579626
rs6536702
rs762679
rs8105767
rs131797
rs56799554
rs2276182
rs17677991
rs28502153
rs4724
rs75664430
rs143190905
rs2967355
rs2763979
rs35446936
SNP
0.00
0.03
0.06
0.09
Effect size (b)
